# Using a learning needs assessment to develop infection prevention training for certified nursing assistants

**DOI:** 10.1017/ash.2022.152

**Published:** 2022-05-16

**Authors:** Erin Garcia, Tisha Mitsunaga, Vikram Haridass, Brieanne Martin, Neha Sardana, Lisa Franqui, Kiya Komaiko, Tracy Lanier, Erin Epson

## Abstract

**Background:** In 2021, the California Department of Public Health Healthcare-Associated Infections Program developed new infection prevention and control (IPC) training for skilled nursing facility (SNF) certified nursing assistants (CNAs), as part of the CDC Project Firstline. CNAs comprise approximately one-third of SNF healthcare personnel (HCP) nationwide; ~50,000 CNAs are employed in California SNFs. Despite making up a large proportion of direct care HCP, CNAs can frequently lack understanding of fundamental IPC practices, including hand hygiene and appropriate personal protective equipment use. We conducted a learning needs assessment for SNF can and leadership to understand and design our program to mecanCNA IPC training needs and preferences. **Methods:** We distributed the learning needs assessment via SurveyMonkey in English and Spanish with questions regarding current IPC practices and challenges, as well as preferred training delivery methods and posttraining support. We leveraged partnershipscanth CNA-affiliated organizations to engage CNAs throughout California. **Results:** Of 193 respondents, 80 (41%) were CNAs and 113 (59%) were leadership staff, representing 97 SNFs in 41 local health jurisdictions. Among CNAs, 34 (43%) believed that they had to do workarounds in their IPC practice and 18 (23%) stated that they would benefit from one-on-one question-and-answer sessions with an infection prevention expert. Also, 50 (63%) selected visual learning, 34 selected (43%) in-person learning, and 30 (38%) selected live or online trainings as their preferred learning style and training method. Most CNAs stated that they were most comfortable listening and speaking (73%) and reading (76%) in English only, followed by listening and speaking (16%) and reading (13%) in English and Spanish. For posttraining support, CNAs preferred access to online training materials (75%), digital materials (68%), virtual office hours with IPC educators (53%), and regular webinars (49%). **Conclusions:** The results of our learning needs assessment confirm the need for accessible IPC training and materials and continued engagement with posttraining support for CNAs. We will continue to provide online training and resources, access to IPC experts including an ‘AskBox’ for CNAs to e-mail IPC questions or request one-on-one support, and monthly office hours. Even though most CNAs are comfortable with training in English only, we will translate curricula into Spanish to support our bilingual Spanish-canaking CNA population. We are developing a tool kit to support SNFs and local health jurisdictions interested in providing their own training using our materials, and we will offer icanerson CNA training. We will use our experience from this process in future learning needs assessments to inform other frontline HCP training, including for SNF environmental services staff.

**Funding:** None

**Disclosures:** None

